# The dual role of cellular senescence in human tumor progression and therapy

**DOI:** 10.1002/mco2.695

**Published:** 2024-08-19

**Authors:** Liang Ma, Jie Yu, Yidian Fu, Xiaoyu He, Shengfang Ge, Renbing Jia, Ai Zhuang, Zhi Yang, Xianqun Fan

**Affiliations:** ^1^ Department of Ophthalmology Ninth People's Hospital Shanghai JiaoTong University School of Medicine Shanghai China; ^2^ Shanghai Key Laboratory of Orbital Diseases and Ocular Oncology Shanghai China

**Keywords:** cellular senescence, cGAS–STING, SASP, senolytic, therapy‐induced senescence

## Abstract

Cellular senescence, one of the hallmarks of cancer, is characterized by cell cycle arrest and the loss of most normal cellular functions while acquiring a hypersecretory, proinflammatory phenotype. The function of senescent cells in cancer cells varies depending on the cellular conditions. Before the occurrence of cancer, senescent cells act as a barrier to prevent its development. But once cancer has occurred, senescent cells play a procancer role. However, few of the current studies have adequately explained the diversity of cellular senescence across cancers. Herein, we concluded the latest intrinsic mechanisms of cellular senescence in detail and emphasized the senescence‐associated secretory phenotype as a key contributor to heterogeneity of senescent cells in tumor. We also discussed five kinds of inducers of cellular senescence and the advancement of senolytics in cancer, which are drugs that tend to clear senescent cells. Finally, we summarized the various effects of senescent cells in different cancers and manifested that their functions may be diametrically opposed under different circumstances. In short, this paper contributes to the understanding of the diversity of cellular senescence in cancers and provides novel insight for tumor therapy.

## Graphical abstract

The study of cellular senescence is a long process, and people continue to explore new mechanisms to improve the role of cellular senescence in cancer, and now we can judge that cellular senescence plays different roles in a variety of cancers, and its duality in cancer requires the attention of doctors and researchers.

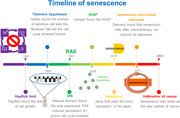

## 1 INTRODUCTION

**FIGURE 1:**
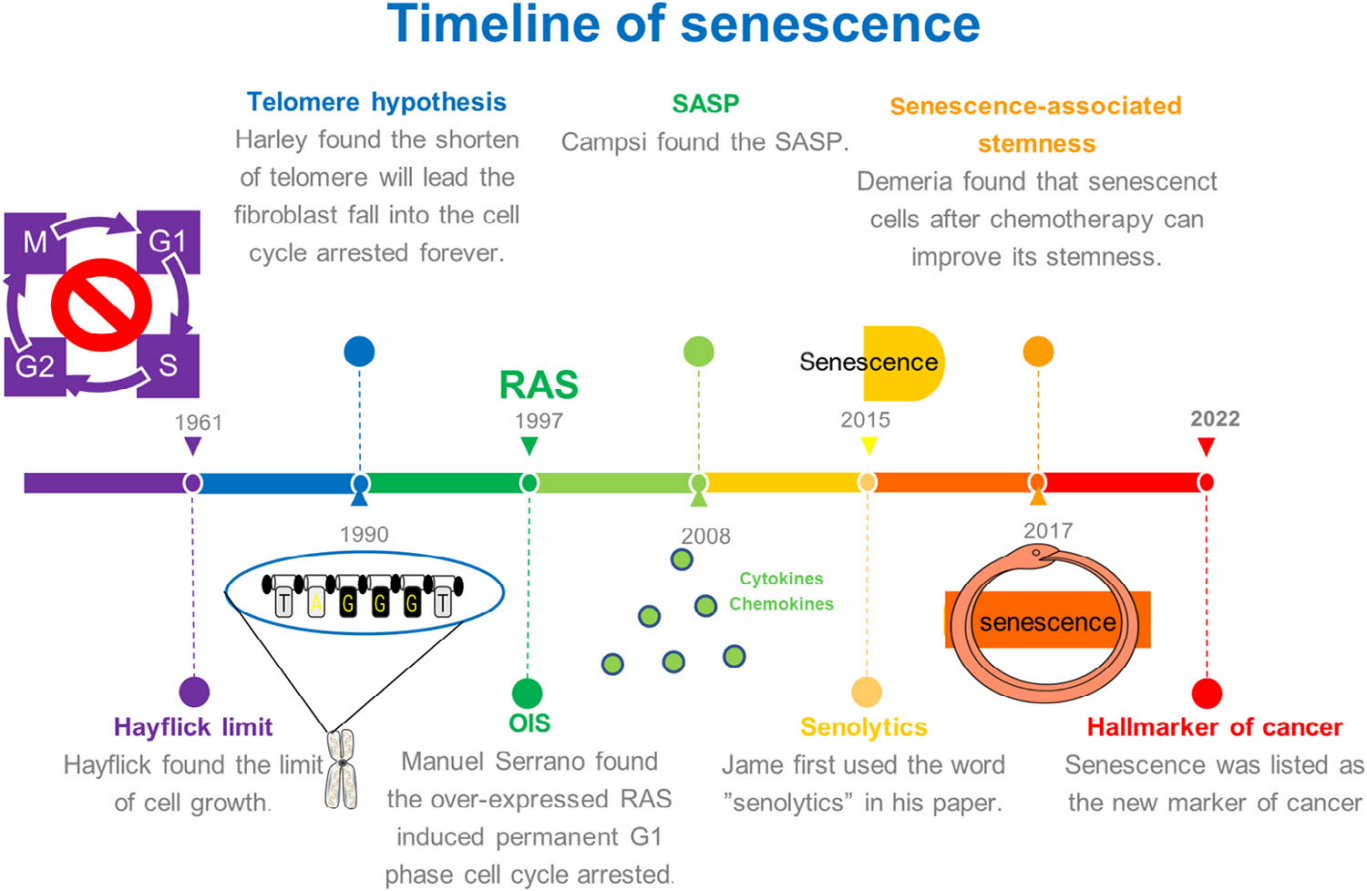
Timeline of cellular senescence. Timeline of the history of senescence research, since the birth of the Hayflick limit in 1961, the introduction of the telomere hypothesis in 1990, the introduction of the concept of SASP in 2008, the introduction of the concept of senolytics in 2015, and the discovery of the stemness of senescent tumor cells in 2017, until 2022 senescence is listed as one of the four new hallmarks of cancer, senescence research has gone through six decades.

In 1961, Leonard Hayflik discovered the phenomenon of cellular senescence when the fibroblasts cultured in the laboratory had a limited ability to split—a phenomenon now known as the “Hayflick limit.”^1^ “Hayflick limit” was doubted due to lack of strong evidence to support it at that time, but this does not diminish its great significance. In the 1990s, Harley reported that telomere shortening led fibroblasts to undergo permanent cell cycle arrest, and in 2003, telomere shortening was verified as the cause of cellular senescence in the same cells in which it was initially identified.^2^, ^3^ In 1997, Manuel Serrano reported that the overexpression of RAS triggered the premature senescence,^4^ which is now acknowledged as the discovery of oncogene‐induced senescence (OIS). In 2008, the molecular biologist Judith Campisi discovered the senescence‐associated secretory phenotype (SASP), noting that interleukins (ILs) secreted by senescent cells affect neighboring cells, inciting local inflammation, increasing the burden on the immune system, and promoting the occurrence of cancer. In addition to two other research groups, these researchers found that senescent cells secreted large quantities of cytokines, growth factors, and proteases, all of which exerted effects on neighboring cells.^5^, ^6^, ^7^ In the same year, Deursen discovered p16lnk, a key gene that promotes cellular senescence,^8^ and verified its expression in collaboration with Professor James Kirkland in 2011.^9^ Moreover, these scientists showed that senescent cells expressing p16lnk4a were cleared from mice, delaying or preventing tissue dysfunction.^9^ In 2015, James Kirkland first used “senolytics” in reference to a class of drugs that cleared senescent cells.^10^ In 2017, Schmitt reported the induction of senescent cells by chemotherapy, which explained the reduced effect of therapy‐induced senescence (TIS) over time.^11^ In 2022, senescent cells were acknowledged as a hallmark of cancer, a milestone in research into the status of cellular senescence in cancer.^12^ Herein, we elaborated on the key aspects of cellular senescence in cancer and explained senescent cells in detail (Figure 1).

Senescent cells are a highly heterogeneous biological phenomenon that play different roles in different situations. Senescent cells are present throughout a human's life and increase significantly in old age. According to the evidence obtained to date, senescent cells are produced in the embryonic development stage, and senescent embryonic cells are not cleared by the immune system but are retained into the postnatal period, with some senescent cells becoming normal cells in neonates, suggesting that cellular senescence is not irreversible.^13^ Senescent cells in the embryonic stage play a role in shaping tissue, and senescent cells in the normal human body promote the healing of damaged tissues and can prevent cancer,^14^ and cells have to bypass this senescence barrier to become carcinogenesis. However, when cells become carcinogenesis, senescent cells in cancer play a procancer role. Senescent cells can help cancer spread because of a process called SASP, which is proven to encourage nearby tumor cells to move and grow, and it can also disrupt immune cells.

The factors that cause cellular senescence are numerous and complex, but both tumor senescence and tumor microenvironmental cellular senescence ultimately affect the cell cycle through the p53/p21 or p16 pathway.^15^ We introduced five major senescence‐inducing factors: DNA damage response (DDR),^16^, ^17^ telomere attrition,^18^, ^19^ reactive oxygen species (ROS),^20^, ^21^ epigenetic,^22^ and mitochondrial dysfunction associated with senescence (MiDAS).^23^ DDR is commonly caused by various physical and chemical factors, such as ultraviolet light,^24^ radiation, chemotherapeutic drugs, and so on, and it is one of the main modes of TIS. Telomere wear is the earliest hypothesis explaining the phenomenon of senescence, and it has been found that telomere attrition‐induced senescence is related to both ROS and DDR; ROS causes senescence through direct damage to DNA, which is commonly seen in various inflammatory responses.^21^, ^25^ The study of epigenetics and senescence has recently attracted a great deal of attention, as epigenetics appears to be the key to senescence reversal,^26^, ^27^ which also essentially affects the cell cycle by altering gene expression; MiDAS, on the other hand, arises from mitochondrial damage, and the mitochondria of senescent cells are usually in an abnormal state, leading to the production of ROS in association with DDR. Targeting different senescence‐inducing factors and thus selecting drugs for tumor treatment is the key to tumor senescence therapy.

Senolytics are a key type of treatment that targets and removes senescent cells. They have shown some positive effects in treating tumors by getting rid of these senescent cancer cells. We introduce the most common senescence drugs in the context of the clinic: the activation of B‐cell lymphoma (BCL) inhibitors,^28^ flavonoid,^29^ NA+/K+ ATPase,^30^ adenine nucleotide transpose 2 (ANT2) inhibitors,^31^ Forehead box O4 (FOXO4) inhibitors,^32^ Heat shock protein (HSP) 90 inhibitors,^33^ and the most commonly used SASP inhibitors—mTOR inhibitors, as well as the CAR‐T therapy and gene editing in senescence. The prospect of senescence scavengers in oncology is still being explored, and there are currently no marketed senescence scavenging drugs for either oncological diseases or not.

By listing six tumors with high correlation with senescence: leukemia,^34^ melanoma,^35^ pancreatic cancer,^36^ breast cancer,^37^ prostate cancer,^38^ and lung cancer,^39^ we describe in detail the senescence evasion phenomenon of tumor cells in each cancer and the effects of tumor senescence, thus enabling readers to visualize the relationship between senescence and cancer from a clinical perspective. As a recently acknowledged cancer marker, cellular senescence is worthy of further exploration by cancer researchers. We present this review to help readers obtain a preliminary understanding of senescent cells, including their dual functions in cancer, in addition to the main cell signaling pathways that play a role in cellular senescence and the treatments available for cellular senescence. We believe that cellular senescence is a stumbling barrier to successful cancer treatment; therefore, we contend that effectively preventing the negative effects of cellular senescence should be the focus of cancer treatment in the future.

## 2 CHARACTERISTICS OF CELLULAR SENESCENCE

### 2.1 The occurrence of cellular senescence

**FIGURE 2:**
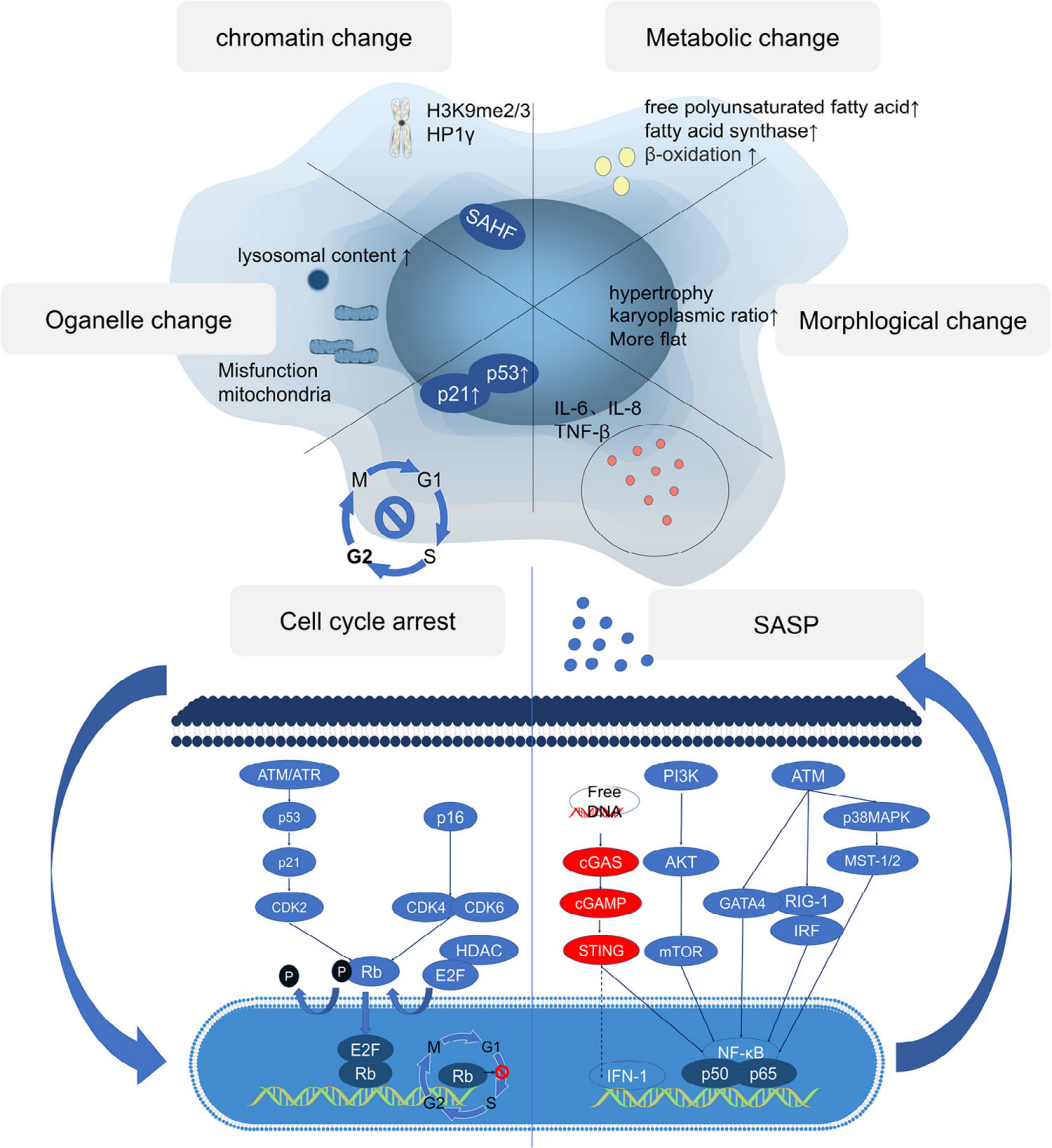
Classical mechanisms of cellular senescence. Senescent cells have unique characteristics and are known as zombie cells; It has six main features: cell cycle stagnation; SASP; organelle changes; changes in nuclear chromatin; metabolic changes; cosmetic changes. The cycle blockade of senescent cells is mainly due to the activation of ATM/ATR caused by DNA damage and the activation of p16ink4a, which are the main ways of senescence. By activating the cell cycle arrest protein, respectively, the Rb protein is dephosphorylated, and the replication of cellular DNA is prevented after binding to the E2F transcription factor, and the cell cycle is stagnant in the G1 phase.

When cells enter the senescent state, the expression levels of the antiapoptotic genes BCL‐W, BCL‐2, and BCL‐XL substantially increase, which promotes cellular antiapoptotic properties. For most senescent cells, two pathways are known to lead to cell cycle arrest—the p16/Rb and p53/p21 axes. In the p16Ink4a/Rb pathway, upon activation of the INK4a/ARF genetic locus, the protein p16Ink4a, a cyclin‐dependent kinase inhibitor (CDKI), directly inhibits the CDK4–cyclin D complex, which allows the dephosphorylation and stabilization of the Rb–E2F complex and thus the inhibition of the cell cycle. In the p53/p21 pathway, p53 is activated via phosphorylation (p‐p53) after cell exposure to a DNA‐damaging stimulus and subsequently upregulates the transient expression of the CDKI p21CIP1. p21CIP1 inhibits CDK2–cyclin E, which allows the dephosphorylation of Rb, leading to the sequestration of E2F and thus cell cycle arrest (Figure 2).

Upon cells became senescent, they would undergo dramatic changes in function and morphology, and almost lose the functions of normal cells. The morphology of senescent cells changes significantly, as indicated by an abnormal increase in the karyoplasmic ratio, flatness, and hypertrophy. These changes are not merely the result of cellular senescence. In contrast, they are driving forces that promote cellular senescence. When the volume is abnormally increased, a cell is unable to increase nucleic acid levels or the protein biosynthesis rate to meet the demands of the larger cell; therefore, the cytoplasm is diluted, leading to cellular senescence.^40^ In addition, senescent cells have altered metabolism, with elevated fatty acid and lactate functions, dramatic changes in chromatin conformation and heterochromatin formation, abnormal organelle function and composition, a significant rise in SA‐β‐gal in lysosomes and mitochondrial dysfunction, cell cycle blockage and cessation of division under the action of Rb, and activation of transcription factors, such as NF‐κB and AMPK in senescent cells, which secrete large amounts of SASP.

### 2.2 The SASP

Senescent cells secrete many cytokines, matrix remodeling enzymes, and so on, which are collectively known as SASP. The regulation of SASP relies on NF‐κB; in addition to regulating its classical pathway PI3K/AKT/MTOR, as well as ATM and its downstream targets, as well as the newly discovered cGAS–STING (a pathway involved in innate immune response) are also involved in the regulation of SASP, which is activated by the increased intracytoplasmic free DNA in senescent cells. In addition to activating NF‐κB, it can also directly promote the transcription of IFN‐1.

The SASP may be the most important feature of cellular senescence and includes IL‐8, IL‐6, and TNF‐β. The major pathway modulating the SASP is NF‐κB, but we also elaborate upon the latest research on cGAS–STING.

First, scientists believe that the SASP regulates IL6/8 expression. Notably, MCP1 and enzymes have been shown to remodel the extracellular matrix in an IL1‐dependent manner. Researchers subsequently identified other SASP‐regulating factors, such as CCAAT/enhancer‐binding protein β (C/EBPβ), which is involved in transcriptional regulation, p38 MAPK,^41^ RIG‐1,^42^ and mTOR,^43^ all of which activate NF‐κB. Moreover, when NF‐κB was knocked down, the SASP disappeared, but other cellular senescence phenotypes did not change, indicating that the NF‐κB pathway independently regulated the SASP.^44^, ^45^ And cGAS can regulate SASP by activating NF‐κB,^46^, ^47^ knocking down cGAS abolished the SASP phenotype, and the prognosis of lung adenocarcinoma patients with low cGAS expression was poor.^48^ cGAS–STING is a signaling pathway comprising the second messenger cyclic GMP–AMP (cGAS) and the cyclic GMP–AMP receptor stimulator of interferon genes (STING). The innate function of cGAS–STING in the regulation of antiviral and inflammatory cytokines and chemokines. As a DNA receptor, cGAS releases the secondary messenger cGAMP to activate STING, which in turn activates downstream genes, inducing IFN1 to promote the expression of SASP genes. Interestingly, cGAS is activated by heterochromatin and constitutively inhibited in the normal nuclear environment, in which abundant chromatin is present.^48^ It has been reported that DNA damage markers may be keys to cGAS activation mediated by extracellular DNA fragments^49^ (Figure 2).

**FIGURE 3:**
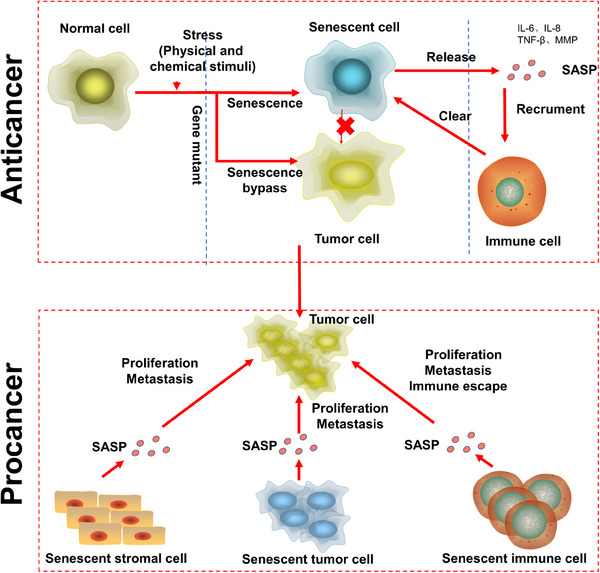
The dual function of cellular senescence in cancer. Senescence is a barrier before normal cells become carcinogenesis after stimulation. After stimulation, cells become senescent and lose their ability to proliferate, do not become carcinogenesis, and secrete SASP to recruit immune cells to clear themselves. However, in the case of a specific gene mutation, the cell undergoes senescence evasion after stimulation and becomes carcinogenesis. In carcinogenesis tissues, the presence of senescent cells for various reasons generally promotes tumor development. Senescent stromal cells create an inflammatory environment to promote tumor proliferation and metastasis through SASP; senescent tumor cells themselves have the same effect, and the stemness of senescent tumor cells increases, which can easily lead to tumor recurrence; senescent immune cells not only secrete SASP to promote tumor proliferation and metastasis, but also lose their immune function. Senescent immune cells not only secrete SASP to promote tumor proliferation and metastasis, but also lose their own immune function due to senescence, leading to tumor immune escape.

In senescent cells, due to the lack of laminB1, heterochromatin fragments leak from the nucleus, and the DNA fragments that accumulate in the cytoplasm bind to cGAS and activate cGAS–STING to regulate the SASP.^50^ Recent evidence has shown that cGAS–STING can regulate TLR2 activity in OIS and promote the secretion of SASP components. Inhibition of cytoplasmic DNase 2/TREX1 contributed to cytoplasmic DNA accumulation in a mouse liver cancer model and promoted the expression of SASP genes, and blockade of cGAS–STING binding to cytoplasmic DNA inhibited SASP acquisition.^51^ The mechanism by which intracellular DNA activates cGAS is unclear, but studies have shown that *line‐1*, a retrotransposable element in primates that has been retained in humans, promotes and maintains the secretion of SASP proteins. Interestingly, *line‐1* has been shown to be inhibited in normal young people. Moreover, the reverse transcription product of line‐1 translated from cDNA accumulated in senescent cells.^52^ Some studies have reported that a deficiency in brain and muscle ARNT‐like protein 1 (BMAL1) causes L1‐induced senescence in primate mesenchymal stem cells and mouse models, proving that BMAL1 is a potential heterochromatin stabilizer.^53^, ^54^ Surprisingly, the STING–PKR‐like endocrine retina kinase (PERK)–EIF2α pathway has been found to be very important for the occurrence of cellular senescence. Notably, inhibition of this pathway significantly attenuates lung and kidney fibrosis, which indicates that the role of cGAS–STING in cellular senescence involves more regulatory mechanisms than that mediating SASP gene expression.^46^ These complicated roles of cGAS–STING may include the induction of IFN1 by cGAS–STING. IFN1 can promote the production of ROS, which are important for cellular senescence.^50^ In general, the cGAS–STING pathway can promote the production of SASP proteins, thereby inducing the recruitment of immune cells and the elimination of senescent cells. These cGAS–STING pathway functions explain why cancer patients with low cGAS expression exhibit poor prognoses. Notably, when immune cells cannot eliminate senescent cells, the long‐term release of SASP proteins exerts a negative impact (Figure 3). In particular, STING is related to multiple proinflammatory genes in cancer cells. When senescent cancer cells cannot be removed, the resulting inflammation leads to poor prognosis.^51^

### 2.3 The heterogeneity of cellular senescence

Senescent cells play contradictory roles in the human body, and their functions are usually related to the antiagonist pleiotropy hypothesis. Senescent cells form an early barrier to tumorigenesis and participate in embryonic development and tissue remodeling, and they cause a series of cellular senescence‐related diseases, including cancer, under certain conditions. The antiagonist pleiotropy hypothesis holds that in the process of human evolution, genes beneficial to human beings in the early stage of development are inherited continuously, but genes that tend to harm human beings in their later years are not inherited. The most direct example of this contradictory evolutionary process is the SASP. The SASP is a fixed but personalized feature of cellular senescence. While senescent cells secrete certain SASP proteins, different cell types and types of stress leading to cellular senescence affect the specific conditions of SASP acquisition. These features, in particular, are consistent with the relationship of NF‐κB to complex regulatory mechanisms. For example, inhibiting NF‐Κb‐related IL‐1 secretion does not affect the secretion of other SASP proteins.^55^

Cellular senescence usually is divided into acute and chronic senescence rather than precisely categorize it according to different tissue or cell specificities. The key reason for this rough classification is a lack of suitable markers for senescent cells. Currently, the commonly used marker of cellular senescence is SA‐β‐gal, which is not sufficient for distinguishing various cellular senescence characteristics. Acute cellular senescence occurs mainly after sudden stimulation, for example, tissue repair,^56^ developmental processes,^57^ or oncogene mutation. Its basic physiological role is to activate and recruit surrounding immune cells to eliminate unnecessary cells through the release of SASP factors to drive tissue remodeling.^58^ Chronic cellular senescence is different from acute cellular senescence. It occurs through a gradual increase in stimulation, is not cell specific, and clears cells at random. In particular, chronic cellular senescence is usually harmful and closely related to certain age‐related diseases and cancers.^9^ We think that the key difference between acute cellular senescence and chronic cellular senescence lies in the rate of senescent cell clearance, that is, how the SASP affects immune cells and stomal cell. The immune microenvironment shaped by the cellular senescence of stromal cells promotes tumorigenesis, and immune cells that eliminate senescent cells are inhibited in this microenvironment. Senescent cells prevent tumorigenesis, but when senescent cells are not removed, they cause chronic inflammation in localized tissues and promote tumorigenesis.^59^

How do senescent cells escape clearance? The answer is related to the recruitment of immune cells by SASP factors. When the transcription factor NF‐κB, which is critical for SASP factor secretion, is inhibited, senescent cells cannot recruit immune cells or otherwise cannot be removed.^45^ Recently, a study revealed that when hepatocellular carcinoma cells lacked the CCL2 receptor (CCR2), immune cells associated with the SASP did not eliminate senescent cells, which led to the immune escape of senescent cells and the occurrence of liver cancer.^60^ Some researchers have hypothesized that this outcome is related to the mechanism by which senescent cells promote tissue healing. When a tissue is damaged, senescent cells maintain the integrity of the tissue and are not eliminated.^61^

In the short term, the SASP induces immune cells to clear senescent cells. However, when recruited immune cells cannot efficiently clear senescent cells to restore normal tissue function, the SASP leads to negative effects, such as senescent cell‐induced tissue fibrosis caused after radiotherapy^62^ and cancer treatment‐induced senescent cell promotion of cancer recurrence and metastasis.^63^

The idea that cellular senescence is a stable and irreversible cellular state was the prevalent theory for a long time. However, abundant evidence has shown that cellular senescence can be reversed via this process.^64^ For example, researchers introduced mitochondrion‐targeted hydrogen sulfide into human vascular endothelial cells and found that this treatment reduced the number of senescent cells by 40–50%, essentially reversing not only the cellular senescence phenotype but also the shortening of telomere length in senescent cells without increasing the degree of DNA damage.^65^ In 2015, *Cell* published an article showing that hydrogen sulfide can regulate life processes as a signaling molecule,^66^ but the mechanism underlying the reversal of the senescent cell phenotype by hydrogen sulfide is unclear. The underlying mechanism may be related to the ability of hydrogen sulfide to prevent free radicals from attacking mitochondria, thereby delaying cellular senescence.

Senescent cells are highly heterogeneous, and this heterogeneity includes differences in the expression of genes. For example, In normal senescent cells, both p21 and p16 are elevated, whereas p21 is highly expressed in senescent endothelial cells, whereas p16 will not significantly change.^67^ SA‐β‐gal is expressed in the vast majority of senescent cells but is consistently highly expressed in lysosome‐overexpressing normal cells, such as macrophages.^68^ Differences in senescent cells depend on the types of cells and stresses that cause cellular senescence and on gene expression in a single senescent cell that relies on temporal dynamics. All these factors make it difficult to find a universal and highly specific marker for senescent cells.^62^

## 3 CELLULAR SENESCENCE INDUCERS IN CANCER

**FIGURE 4:**
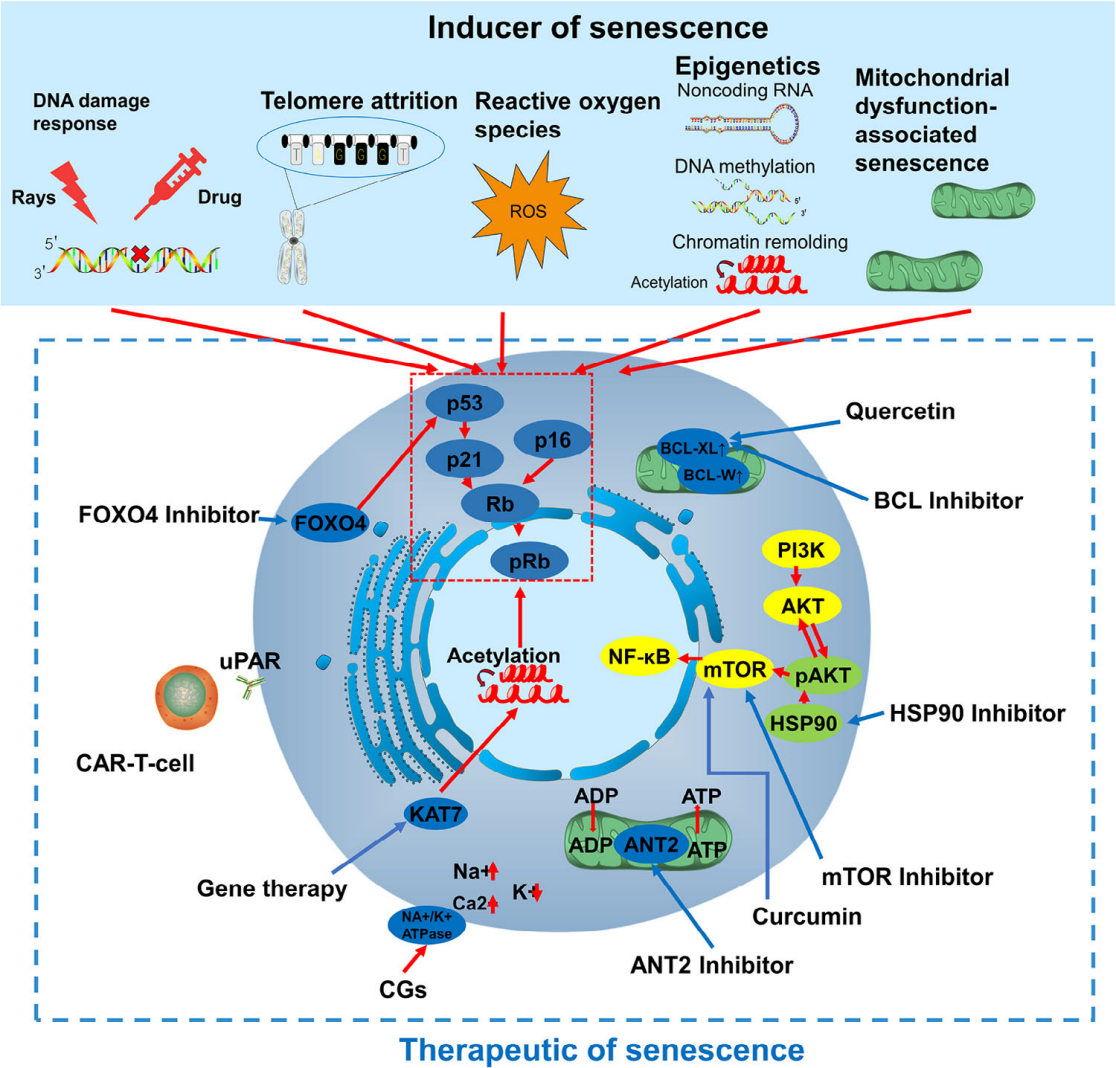
The mechanism of inducer of senescence and therapeutics of senescence. (1) Inducer of senescence: DNA damage response; telomere attrition; reactive oxygen species; epigenetics; MiDAS. The above senescence‐inducing factors intersect with each other, and they all induce senescence by affecting p53/p21 or p16. (2). Therapeutics of senescence: quercetin: acts on BCL2 family proteins, inhibits BCL2 family proteins to cause mitochondrial functional state changes and induces apoptosis in senescent cells; HSP90 inhibitor: inhibits the protective effect of HSP90 on phosphorylated AKT and inhibits mTOR activation; mTOR inhibitor: acts on mTOR and inhibits mTOR activation; mTOR inhibitor: acts on mTOR and inhibits mTOR activation. HSP90 inhibitor: inhibits the protective effect of HSP90 on phosphorylated AKT and inhibits the activation of mTOR; mTOR inhibitor: inhibits mTOR and inhibits the NF‐κB pathway; ANT2 inhibitor: inhibits the ADP/ATP exchange responsible for ANT2, inhibits glycolysis, and inhibits energy supply of senescent cells; CGs: inhibits Na+/K+ATPase, and promotes the inward flow of Ca2+ leading to the imbalance of cellular homeostasis; Gene therapy: utilizes the gene editing method represented by CRIPSR Gene therapy: targeting the key senescence protein KAT7 by crispr9 gene editing technology to inhibit chromosome acylation and gene transcription; CAR‐T: removing senescent cells by targeting CAR‐T cells that specifically express the receptor u‐PA in senescent cells; FOXO4 inhibitor: removing senescent cells by blocking the interaction between FOXO4 and P53.

During cellular senescence, cell cycle progression in cells exposed to a series of nonlethal stimuli is permanently stopped, rendering the cells highly resistant to apoptosis. There are many factors that lead to cellular senescence; according to the physiological changes in senescent cells, they can be divided into telomere shortening, gene mutation, drug stimulation, physical stimulation (Figure 4), and so on. In addition, senescence induced in tumor patients due to physical and chemical stimuli of the treatment process is specifically referred to as TIS. TIS mainly rely on the DNA damage induced by p53 protein activation^69^; however, recent studies have revealed that TIS is also deeply linked to epigenetics may provide new ideas for senescent treatments.^70^

The following attributes can be categorized on the basis of the stimulus that induces senescence.

### 3.1 DNA damage response

DNA strand breaks caused by sunlight in nature, chemicals, or radiation from cancer treatments can cause DDR in cells. When DNA damage reaches a certain level, cells undergo cellular senescence. DDR‐induced senescence depends on the p53 pathway, which stimulates the DDR to activate ATM/ATR kinase and downstream p53; upregulates p21; activates CDK2, a cell cycle inhibitor; and inhibits cyclin E to stall cells in the G1 phase of the cell cycle. A plethora of evidence proves that a lack of p53 can reverse DDR‐induced senescence.^71^, ^72^

Part of the approach to treating cancer patients is to directly target the DNA replication process in cancer cells, especially since the high‐energy rays utilized in radiotherapy and chemotherapeutic drugs such as platinum and adriamycin can also directly lead to DNA strand breaks, causing DDR‐induced cellular senescence. DDR senescence is important for tumor recurrence and progression, and removal of senescence induced during radiotherapy is important for patient prognosis.^73^

### 3.2 Telomere attrition

Telomere loss is irreversible in somatic cells because this process prevents chromosome fusion. The suppression of chromosome fusion is the basis of the anti‐DNA repair properties of telomeres, and the DNA repair system cannot repair telomere damage.^74^ It has long been accepted that impaired telomere function alone can cause cellular senescence; however, recent studies have revealed that telomeres may cause cellular senescence because of DNA damage. Rodier's team performed experiments that suggested that cellular senescence, which occurs when cell replication stops, is rooted in irreversible damage to genes, not simply telomeres.^75^ Moreover, damaged genes may cause cells to enter a presenescent state, and when the damage to telomeres is rapidly reversed, genomic damage and cellular senescence can be prevented. By introducing telomerase to vascular endothelial cells in vitro, Mojiri and colleagues^76^ showed that the reversal of cellular senescence phenotypes was mediated by attenuated telomere damage.

OIS is the result of oncogene mutations leading to replication pressure and telomere attrition^77^; however, in the case of telomerase activation, cells with mutated oncogenes will enter a state of unlimited proliferation, at which time, if appropriate external stimuli are applied to the cancer cells, such as radiotherapy, chemotherapy, and so on, the activity of telomerase decreases, and the cancer cells will reactivate the oncogenes, such as p53, p21, and so forth, and will enter a state of senescence.^78^, ^79^ The development of telomere theory today has encountered many obstacles. For example, there is no obvious connection between senescence and telomere length in mice.^80^ A clear link between telomerase deficiency‐induced telomere loss and senescence has been observed in some human cells, but telomerase expression is observed in human hematopoietic stem cells with telomeric DNA loss.^81^ These perplexing questions have not been resolved.

### 3.3 Oxidative stress

ROS are common senescence triggers and intersect with a variety of other triggers, include DDR,^82^ telomere attrition,^83^ epigenetic modifier activity,^84^ or MiDAS,^85^ among others. The essence of the role of ROS in all of the above pathways stems from the powerful oxidizing ability of its ROS. Inhibiting the ROS component in cells is key to preventing ROS‐induced senescence.

Natural antioxidant mechanisms exist in cells, Nrf2 is a key transcription factor that drives antioxidant gene expression^86^; however, Nrf2 is generally inactivated in tumor cells.^87^, ^88^ When Nrf2 is downregulated, the antioxidant capacity of cells is reduced, exacerbating DNA damage and causing cellular senescence.^89^, ^90^ Radiation therapy for cancer depends on the killing effect of ROS produced by radiation.^91^ And ROS are necessary for radiation‐induced tumor cell senescence.^92^ When a ROS scavenger is used before radiation induction, tumor cells will not undergo cellular senescence.^93^ Tissue fibrosis due to radiation therapy is also associated with ROS‐induced senescence. Cellular senescence induced during radiation therapy promotes tissue fibrosis and the production of ROS, proinflammatory factors, and TGF‐β. The latter three types of factors cooperate to promote tissue fibrosis, and the greater the degree of radiation‐induced senescence is, the more severe the fibrosis.^94^ Therefore, inhibition of ROS‐induced senescence may be the key to improving the prognosis of radiotherapy.

### 3.4 Epigenetic modifiers

Chromatin remolding, histone posttranslational modifications, DNA methylation, and noncoding RNAs cooperate with each other to induce cellular senescence. Replication cellular senescence is closely related to DNA methylation, and experiments have shown that in the absence of epigenetic modification, particularly during the senescence‐associated methylation process, pluripotent stem cells in culture do not undergo cellular senescence and that cellular senescence caused by radiation exposure is not closely related to this process.^95^, ^96^

In cancer, changes in gene expression without altering the DNA sequence (epigenetic changes) can cause cancer cells to senescence. For example, adding methyl groups to DNA can turn on genes that suppress tumors, making the cancer cells enter a senescent state^97^; histone deacetylase inhibitors can promote senescence or apoptosis in cancer cells by increasing the level of acetylation of histones; Certain miRNAs can induce senescence by targeting the mRNAs of oncogenes and inhibiting their expression, and long‐chain non‐coding RNAs can also induce senescence by interacting with histone modification complexes and altering the epigenetic status of genes. In addition to these three most common epigenetic alterations, chromatin remodeling and cell cycle regulation may also be involved in cancer cell senescence. By regulating these epigenetic processes, it is possible to control cancer senescence without altering the gene sequence, and the current finding that cellular epigenetic control of senescence is reversible may lead to a new direction for the future treatment of cancer senescence.

TISs reshape the epigenetic characteristics of certain cancer cells, allowing some cancer cells to acquire stem cell characteristics, thus increasing the probability of cancer recurrence and metastasis.^98^ SAHF plays key roles in TIS. TIS is thought to be characterized by the S‐phase entry‐blocking effect of histone 3 lysine 9 trimethylation (H3K9Me3) and is dependent on the methyltransferase Suv39h1, which is related to heterochromatin formation. The modification of H3K9 by suv39h1 was confirmed to be a newly discovered tumor inhibition mechanism.^99^ Notably, however, TIS did not depend on p53. Generally, the cellular senescence caused by DNA damage is activated through ATM and depends on CDKs. After p53 was knocked out, researchers successfully used doxorubicin to induce cellular senescence.^100^

### 3.5 Mitochondrial dysfunction associated with senescence

Mitochondrial function is interesting in senescent cells. Various cellular senescence mechanisms are triggered by mitochondrial dysfunction. For example, mitochondrial dysfunction can activate RAS gene expression and cause cellular senescence. In addition, the mTORC1 pathway can trigger cellular senescence. The mechanism involves MiDAS activating mTORc1, which in turn activates P70S6K. P70S6K can inhibit HDM2, an inhibitor of p53.^101^ Mitochondrial dysfunction also synergize with oncogene signaling pathways to initiate replicatory cellular senescence.^102^

Mitochondrial functional status is crucial for cancer cells, and mitochondrial damage caused by radiotherapy is also an important cause of cancer cell senescence, and tumor recurrence can be effectively prevented by eliminating radiotherapy‐induced senescence.^103^, ^104^ Mechanical force signals mediated by the cellular matrix also affect the mitochondrial function of cancer cells thereby causing cellular senescence.^105^

Since mitochondria are involved in cellular senescence, the potential effect of metformin needs to be discussed. Metformin treatment is currently very popular and seems to exert an antiaging effect.^106^ Specifically, metformin inhibits oxidative phosphorylation and reduces the amount of ROS produced via aerobic respiration in cells. However, senescent cells do not rely on oxidative phosphorylation processes for energy supply,^107^ metformin may be unhelpful. Some evidence supports this outcome of metformin use.^108^, ^109^ However, many people still have high hopes for its ability to eliminate ROS to reverse senescent cells.^110^

## 4 THERAPEUTIC EFFECTS IN CANCER CELLULAR SENESCENCE

Senescent cells widely exist in cancer patients, including both senescent cancer cells and senescent stroma and immune cells, both of which have an important impact on cancer patients.^73^ Cancer senescent cells are commonly found in cancer patients after treatment; due to the physicochemical stimulation of radiotherapy leading to a large increase in senescent cells in the tumor, these senescent cells promote tumor recurrence, which has a great impact on cancer patients.^111^ A large number of senescent cells are also present in the tumor microenvironment, and there is evidence that tumor endothelial cells are generally senescent in pan‐cancer and are associated with tumor development and metastasis,^112^, ^113^ and the senescence of immune cells is also an important cause of tumor immunity escape.^114^, ^115^ Removal of these senescent cells is of great significance to tumor patients. Currently, the therapeutic means to target senescent cells mainly focus on drug‐targeted therapy, such as senolytics represented by BCL inhibitors and SASP‐regulating drugs represented by mTOR inhibitors, and so on. In addition, cell therapy and gene editing drugs are also available (Figure 4).

### 4.1 Senolytics

The earliest and most mature senescent cell‐eliminating drugs are called senolytics, a term based on the word senor and lytics, which together indicate cellular senescence clearance. On the basis of the types of research and development, senolytics can be divided into two generations: the first generation, which was developed based on bioinformatics analyses, for example, dasatinib, quercetin, fisetin, and so on. The basic screening model for these drugs is to do large‐scale bioinformatics screening among commonly used drugs and natural compounds in the human body, and ultimately obtain dozens of target drugs.^116^, ^117^ The second generation, which was developed based on more traditional methods, including HSP90 inhibitors,^33^ cardiac glycosides (GCs),^118^ and so on, were discovered through high‐throughput screening and detailed mechanistic studies. Here, we summarized the current senolytics in tail (Table 1).

**TABLE 1:** Therapeutic drugs for treating senescence and the underlying mechanisms.

Type	Pathway	Drug	References
BCL inhibitor	BCL‐X/L/2	ABT‐263 (NCT05222984) ABT‐737 (NCT01440504) Venetoclax (NCT03181126) Obatoclax (NCT01563601) Mesylate (A1331852)	^120^ ^121^
Flavonoids	BCL‐2, PI3K/AKT/mTOR	Quercetin (NCT06355037) Curcumin (NCT01917890) Fisetin (NCT05595499)	^128^ ^125^
Cardiac glycosides	Na+/K+	Digoxin (NCT01162135)	^129^
HSP90 inhibitor	pAKT	Geldanamycin (NCT00019708) Tanespimycin (NCT00546780) Alvespimycin (NCT00089271) Pimitespib (NCT05245968)	^33^
ANT2 inhibitor	Glycolysis	MitoTam (EudraCT 2017‐004441‐25)	^132^
FOXO4 inhibitor	MEK/JUK/p53	FOXO4‐DRI (No clinical trial)	^64^
CAR‐T		uPAR (No clinical trial)	^137^

#### 4.1.1 The BCL inhibitor

BCL family proteins enables senescent cells to resist apoptosis and supports their survival in tissues. Notably, three antiapoptotic proteins, BCL‐2, BCL‐w, and BCL‐xl, are highly expressed in senescent cells. ABT‐737 (a cancer‐targeted therapeutic drug), which showed affinity for all three proteins, can effectively remove senescent cells.

In a clinical review, ABT‐263 (An orally active counterpart to ABT‐737) was shown to have beneficial effects on cancer treatment, senescent cell clearance, and cardiovascular disease treatment. The results of ABT‐737 treatment may be attributable to its wide range of molecular targets,^119^ which are not limited to the BCL family and may be the basis of its efficacy in multiple diseases.^120^ Notably, targeted inhibitors of the BCL‐2 family do not clear senescent cells and cause severe neurotoxicity, and inhibitors of BCL‐XL inhibitors, such as A1331852 and A1155463, have been shown to be effective at preventing this deleterious side effect.^121^ Meanwhile, within tumors after chemotherapy, application of BCL inhibitors to remove senescent cells can significantly improve the prognosis of cancer treatment.^122^

#### 4.1.2 Flavonoids

The first discovered natural flavonoid compound, quercetin, acts on PI3K/mTOR, and quercetin has been found to work synergistically with dasatinib to combat the weaknesses of dasatinib associated with cellular senescence.^10^ The combination of dasatinib with the flavonoid quercetin (D plus Q) is expected to be highly effective because of the findings of mouse experiments performed at the Mayo Clinic by Professor Kirkland, which showed that the health and longevity of aging mice improved after treatment with these senolytic agents.^123^ In addition, Professor Kirkland applied this senolytic therapy to 14 patients with pulmonary fibrosis, and the symptoms of these patients were alleviated.^124^ Recently, D plus Q therapy entered phase II clinical trials.

In addition to quercetin, compounds such as curcumin and fisetin have been shown to inhibit the PI3K/AKT/mTOR pathway, suggesting that they can inhibit a variety of cancers, especially melanoma.^125^, ^126^ The flavonoid cellulose improved the health and prolonged the longevity of adult and elderly wild‐type mice.^29^ Research on curcumin as a drug is more mature than that on other compounds. Curcumin inhibits the activation of the damage response at multiple points in the whole repair pathway in senescent cells, and many clinical experiments have confirmed its effectiveness in clinical applications, for example, in anti‐inflammatory and anticellular senescence treatments.^127^ Like other natural biologics, curcumin has problems, such as low targetability, unstable physicochemical properties, and poor solubility, but these problems can be solved via nanomaterial encapsulation and delivery.^128^

#### 4.1.3 Na +/K+ ATPase

CGs constitute a family of compounds with senolytic activity. Senescent cells express more sodium‐potassium pumps compared with normal cells, which confers the ability of CGs to target senescent cells. By targeting the Na+/K+ ATPase pump, CGs induce an imbalance in the intracellular electrochemical gradient, leading to membrane depolarization and acidification. Senescent cells exhibit a slightly depolarized PM and a high concentration of H+, increasing susceptibility to the action of CG. These weak senescent cells can be leveraged for therapeutic purposes to treat tumors and age‐related diseases.^129^ Digoxin, digitoxin, and ouabain have been proven to play roles similar to those of CGs. Because of their low intracellular pH and further decreases in pH, cells are prone to apoptosis.^130^

GC is a broad‐spectrum antisenescence drug that can be used for tumor senescent cell clearance. Treatment with ouabain or digoxin decreased the levels of (CDKN1a), IL‐1β, and IL‐6, suggesting that ouabain treatment resulted in reduced senescence and reduced SASP. In TIS, CGs also reduced radiotherapy‐induced senescent cells.^118^

#### 4.1.4 ANT2 inhibitor

Senescent cells rely mainly on glycolysis for energy. ANT2 introduces ATP into the mitochondrial matrix during glycolysis, a process to which senescent cells are predisposed.^131^ Since glycolysis is strongly dependent on ANT2 and because TGFβ, a SASP protein, exerts a negative regulatory effect on ANT2, TGFβ‐induced decreases in ANT2 content lead to an increase in the sensitivity of senescent cells to the ANT2 inhibitor MitoTam, an analog of tamoxifen, leading to selective clearance of senescent cells mediated via mitochondria.^132^

Radiation causes enhanced glycolysis: AMPK/NF‐κB‐activated radiation‐induced senescence leads to increased glycolysis,^133^ and various cytokines and chemokines increase the effects of radiation to enhance glycolysis, increase lactate levels, and increase the expression of monocarboxylate transporter‐1, which mediates the export of lactate into the extracellular environment.^134^ This suggests that radiation‐induced senescence promotes a metabolic shift that supports cell survival, radiation resistance, and tumor recurrence. Considering the excellent effect of ANT inhibitors in blocking glycolysis, targeting senescent cell clearance after radiotherapy may be the most appropriate scenario for ANT inhibitors, although evidence corresponding to ANT2 inhibitors for treating senescence in radiotherapy patients is lacking.^135^

#### 4.1.5 FOXO4 inhibitor

FOXO4 is a transcription factor that is activated by phosphorylation via the MEK–JUK pathway and activating p53. p53 activation can transcriptionally activate the downstream gene p21, p21 activates Rb protein to block the cell cycle.^136^ Baar et al.^64^ designed a FOXO4 peptide that selectively targeted p53‐dependent senescent cells, leading to failure of p53 activity in the nucleus, preventing p53‐induced senescence gene activation, and triggering the intrinsic apoptosis pathway. Moreover, this peptide can reduce the negative effects of doxorubicin‐induced FOXO4 upregulation, reduce the hepatotoxicity and weight loss associated with chemotherapy, and reduce the number of chemotherapy‐induced senescent cells.^64^

#### 4.1.6 HSP90 inhibitors

HSP expression is increased in senescent cells. HSP90 can maintain pAKT stability, and pAKT can inhibit apoptosis. When an inhibitor abolishes either of these effects, senescent cells are increasingly prone to apoptosis.^33^

HSP90 inhibitors seem to perform well as senolytics in the laboratory. However, the key and unacceptable problem is that no successful phase III clinical trials of HSP90 inhibitors have been performed for decades; therefore, no useful HSP90 inhibitors are available on the market. Thus, whether HSP90 inhibitors can play a role in the cellular senescence of cancer cells remains unknown.

### 4.2 SASP regulators

Notably, rapamycin does not directly clear senescent cells, but it has significant anti‐SASP effects. Senescent cells exert effects on local tissues through the SASP and enhance the antiapoptotic ability of cells. For example, mTOR can upregulate IL‐1A to promote the translation of SASP mRNAs, and IL‐1A can promote the activity of NF‐κB. Activated NF‐κB subsequently increases SASP gene transcription, and rapamycin inhibits this pathway by inhibiting the translation of IL‐1A in senescent cells, inhibiting the secretion of active substances.^43^ Rapamycin can reduce the formation of senescent cells and decrease the expression of senescence markers.^43^ It can also eliminate senescent cells directly. Combined inhibition of mTORC1 and mTORC2 reverses senescence in presenescent cells, making it a viable treatment option.^138^ However, the side effects of rapamycin in the clinic, including immunosuppression, lipid disorders, glucose tolerance, and diabetes risk, cannot be ignored.^139^, ^140^ Side effects can be prevented by selectively inhibiting mTORC1, and many new mTOR inhibitors have entered the clinical trial phase.^141^ The novel mTOR inhibitor NR1, which is in the development stage, binds to the mTORC1 activator Rheb and prevents its denaturation from activating mTORC1.^142^ Compound DL001, another new mTOR inhibitor, which is significantly more selective for mTORC1 than rapamycin.^143^

The mTOR inhibitor also developed an effective therapy called “one‐two punch.” A study on liver cancer cells, the cellular senescence inducer TP53, a CDC7 inhibitor, was identified through high‐throughput screening.^144^ After confirming that CDC7 inhibited induced senescence, high‐throughput drug screening was performed to confirm that drugs targeting mTOR cleared senescent cells. Combination treatment with two drugs was shown to significantly inhibit tumor growth and prolong survival in mouse models. By exploiting cellular senescence and bypassing its disadvantages, researchers can be expected to leverage cellular senescence for tumor therapy

### 4.3 Cell therapy

Urokinase‐type plasminogen activator receptor (uPAR) is abundant on the surface of senescent cells, and uPAR‐positive cells exhibit a tissue response similar to that of β‐gal‐positive cells. A 2019 report suggested that uPARs may be targets for CAR‐T cells in ovarian cancer. Amor et al.^137^ used uPAR as a target to generate CAR‐T cells and successfully cleared senescent cells, confirming that uPAR is a potential T‐cell‐targeting senolytic agent.

### 4.4 Gene therapy

Clustered regularly interspaced shortpalindromic repeats (CRISPR) therapy has led to promising results in the field of cellular senescence research. Recently, a team at the Chinese Academy of Sciences used CRISPR to identify the senescence‐associated gene KAT7, which induces senescence by promoting acetylation of histone H3 at K14; Knocking out this gene alleviated the cellular senescence of liver cells and extended the lifespan of mice.^145^ Notably, the KAT gene is necessary for the maintenance of leukemia tumor stem cells, and knocking out KAT7 attenuated leukemia.^146^ These findings suggest that the KAT7 gene may inducing senescent cells by histone acetylation, which may contribute to the maintenance of tumor stem cell activity.

The drugs mentioned above represent the cutting edge of current cellular senescence therapies and help us to understand how cellular senescence therapeutics should be selected in the context of different tumors and therapeutic needs. However, all cellular senescence therapeutic agents are still in the experimental stage, and it is not yet known whether cellular senescence therapies will actually enter clinical use.

## 5 CELLULAR SENESCENCE IN CANCER

**FIGURE 5:**
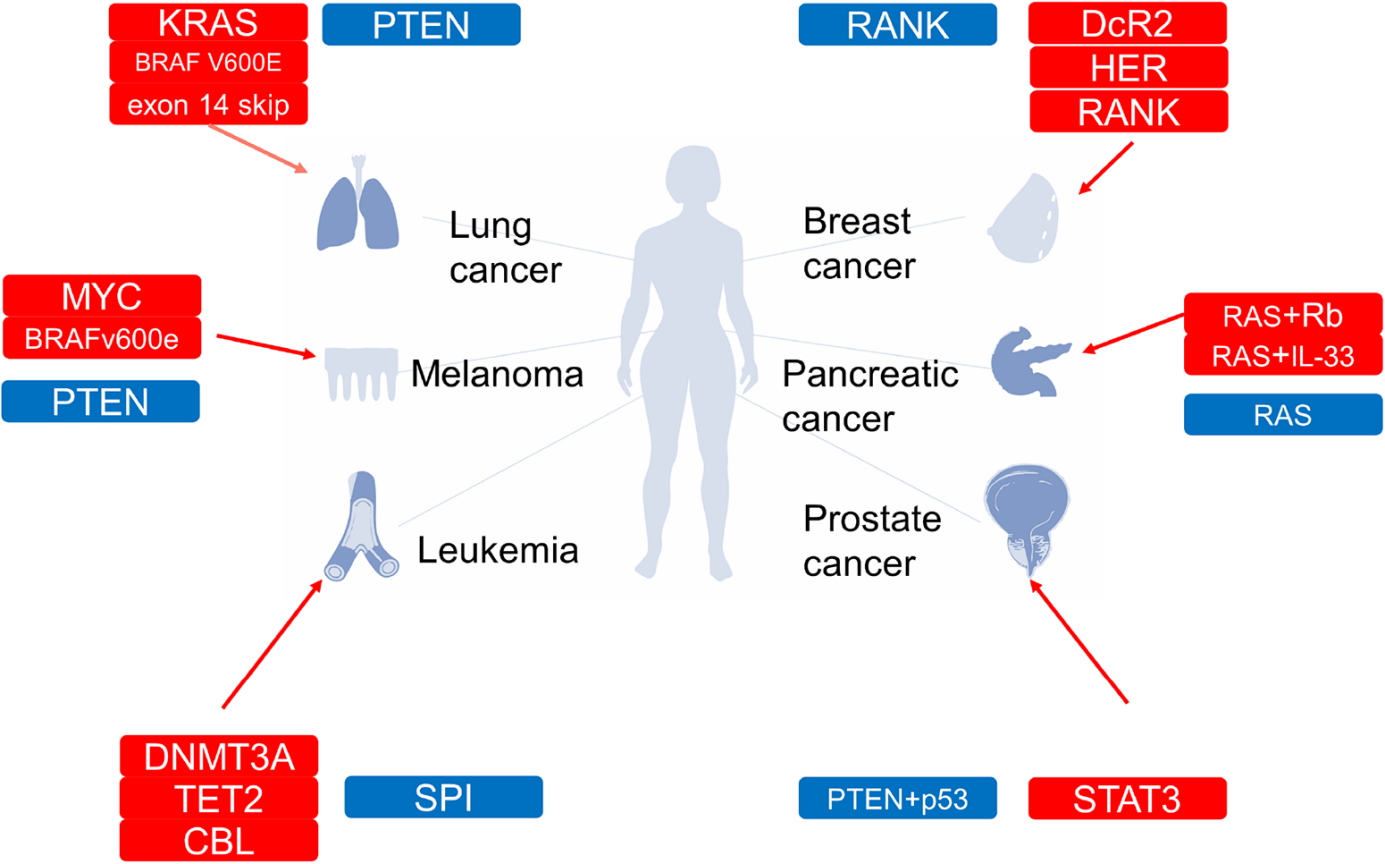
Cellular senescence functions and targets in human cancer. Lung cancer: KRAS mutation, BRAF V600E, and MET exon 14 skip are highly expressed in tissues of aging lung cancer patients, but it is not clear exactly what role these mutations play. MYC loss induces cell cellular senescence and prevents cancer progression; PTEN‐loss population is prone to COPD and thus a potential population for lung cancer. Melanoma: MYC loss induces cell cellular senescence and prevents cancer progression. BRAF mutation alone induces melanoma cell cellular senescence without carcinogenesis, while loss of PTEN causes carcinogenesis in BRAF mutated melanoma cells leukemia: SPI1 is a key gene that prevents hematopoietic stem cell carcinogenesis by cellular senescence; leukemia‐causing mutations represented by DNMT3A, TET2, and CBL are heavily upregulated in aging bone marrow. Breast cancer: RANK protein is a key protein for cellular senescence regulation in breast cancer; before cancer development, RANK is involved in the cellular senescence of breast epithelial cells and prevents cancer development; after cancer development, RANK protein promotes breast cancer development through the cellular senescence of breast stromal cells; DcR2 or HER protein is involved in cancer cellular senescence exemption. Pancreatic cancer: pancreatic RAS mutations alone induce cellular senescence inhibition of cancer in the pancreas, but when RAS mutations are accompanied by mutations in Rb proteins or when there is synergistic effect of IL33, cancer is induced. Prostate cancer: PTEN loss plus P53 loss induces cancer in prostate epithelium avoiding cellular senescence; STAT3 induces prostate cancer cellular senescence and thus inhibits cancer. (*Note*: red, promotes cancer development; blue, suppression cancer development).

When an oncogene is mutated, cells can transform into senescent cells under certain conditions, after which they stop proliferating to inhibit the continued malignant transformation of tissues. However, under the chronic inflammation,^147^ they can promote tumor growth. First, senescent cells exhibit a chronic DDR, leading to continuous DNA damage accumulation. This chronic DDR leads to mitochondrial dysfunction and an increase in ROS, creating feedback loops to help maintain the DDR.^148^ Persistent DNA damage and ROS production trigger intracellular changes that promote malignant transformation. Second, the proinflammatory factors secreted from senescent cells are double‐edged swords. SASP proteins can recruit immune cells to senescent cells to remove senescent cells from tissues, simultaneously promote cellular senescence of surrounding cells,^149^ drive local inflammation, and increase the burden on the immune system. The SASP is a mixture of cytokines, chemical factors, growth factors, and proteases secreted from senescent cells that create an inflammatory microenvironment and tumor microenvironment. The SASP secreted by senescent cells not only maintains their own senescent state but also induces cellular senescence in neighboring cells. In addition, SASP compounds can induce epithelial‐to‐mesenchymal transition and reprogram cancer cells, driving their acquisition of stem cell‐like phenotypes.^150^ Immune cellular senescence refers to age‐related immune system dysfunction, which is caused, at least in part, by the cellular senescence of immune cells and low‐grade chronic inflammation induced by the SASP. The SASP in the tumor microenvironment can drive both inflammation and immune responses, this means that the balance between beneficial and detrimental effects of the immune response is disrupted, favoring harmful outcomes by inducing the accumulation of senescent cells and enabling tumor cell escape from immune responses. This damage to the immune system can also lead to impaired immune monitoring, damage that is essential for tumor progression and metastasis (Figure 5).

Cellular senescence can be targeted to treat cancer through two approaches: reshaping the vascular state and immune environment of tumors through senescent tumor cells and removing the effects of senescent cells by removing senescent cells or blocking the SASP. Senescent cells in different tissues of the same individual exhibit specific traits.^151^ We summarized the mechanisms by which several common oncogenes induce cellular senescence (Table 2).

**TABLE 2:** Oncogenes and suppressor genes associated with senescence.

GENE	State	Pathway	Cancers	References
Oncogene				
RAS	Activation	Raf/MEK/MAPK	Pancreatic cancer	^152^
MYC	Inactivation	p53/p21/Rb	Lung cancer, melanoma	^153^, ^154^
SPI1	Activation	p38MAPK14	Leukemia	^155^
BRAF	Activation	MEK/MAPK	Melanoma, lung cancer	^156^, ^157^
Tumor suppressor gene				
VHL	Inactivation	SKP2/p27/Rb	Renal cell carcinomas	^158^, ^159^
PTEN	Inactivation	mTOR/PI3K/AKT	Prostate cancer	^160^
ING	Inactivation	ITSN2/p16/p57	Lymphomas	^161^

### 5.1 Leukemia

When hematopoietic stem cells encounter a series of stimuli, they undergo cellular senescence and stop proliferating, a process that also prevents hematopoietic stem cells from undergoing carcinogenesis, a defense mechanism present in normal humans.^162^ Transcription factor Spi1 is the key regulator of hematopoietic cell self‐renewal, and in the hematopoietic system, cellular senescence has been shown to be an Spi1‐induced antiproliferative mechanism that can prevent the development of acute myeloid leukemia (AML).^155^ However, via a series of unpredictable events, hematopoietic stem cells escape cellular senescence, inevitably leading to a malignant state. At this point, cellular senescence is a deleterious process. Senescent tumor cells secrete a large amount of SASP compounds that promote malignant cell proliferation. Moreover, proliferating malignant cells undergo cellular senescence, increasing the number of senescent tumor cells and forming a vicious cycle. In addition, senescent cells promote tumor metastasis and increase the possibility of tumor recurrence, indicating that, as scientists, we need to change our thinking about tumor treatment with respect to not only chemotherapy and other methods to combat active tumor cells but also tumor recurrence, as vigilance is needed to prevent the senescent state of tumor cells.^163^

Interestingly, the prevalence of leukemia increases greatly after people reach 70 years of age, and this phenomenon is closely related to the aging of the bone marrow. Specifically, with the cellular senescence of bone marrow hematopoietic stem cells after they are older than 70 years, the ability of bone marrow stem cells to differentiate into lymphocytes decreases greatly, the differentiation of marrow cells increases greatly, and even malignant proliferation occurs.^164^ There is no clear reason for this phenomenon, but many studies have proven that the diversity of hematopoietic stem cell clones after the age of 70 years may suffer “a cliff‐like drop,” and the proportion of clones carrying cancer mutant genes increases dramatically. In this process, IL‐27RA is the executor of bone marrow cell senescence, which triggers the inflammatory reaction in the bone marrow and induces cellular senescence.^165^ Similarly, some researchers have studied the key factor involved in bone marrow cancer, myeloid erythroid transformation‐related gene 3, the level of which determines the balance between the myeloid system and the lymphoid system in bone marrow stem cells.^164^ Recent research from Cambridge showed that mutations that accumulated steadily decades ago were the cause of aging of the bone marrow; these mutations did not occur suddenly but rather accumulated throughout the whole life of this person. Compared with those in young people's bone marrow cells, telomeres in bone marrow stem cells are decreased by 30 pairs of bases per year. Compared with young people's bone marrow, mutated clones continue to proliferate positively, resulting in mutated clones occupying the living space of other clones with slower growth. These driver mutations include DNMT3A, TET2, and CBL, but in fact, these known mutations are relatively infrequent in the abnormally proliferating clones of elderly individuals, which may mean that the root driving genes of blood tumors still need to be explored.^166^

In the field of leukemia treatment, immunotherapy is the most promising option. Researchers are already trying to treat patients with hematologic tumors with CAR‐T; however, in leukemia, CAR‐T has only been shown to be effective against erythrocyte leukemia and B lymphocytic leukemia. Moreover, its use in AML is still being explored.^167^ In addition, CAR‐T cells are effective cellular senescence scavengers, which we describe in detail in the cell therapy subsection. HSCT is an early and successful immunotherapy; however, it cannot be used in people older than 70 years of age, and recurrence is the main reason for the failure of HSCT treatment.^168^ When chemotherapy is used to treat AML, recurrence is still a common problem, even after complete remission. The common belief is that recurrence is caused by AML stem cells. Senescent cells are thought to promote the recovery of stem cell potential.^169^ Duy from the Ari and Fox Chase Cancer Center in the Cornell Medical College confirmed that AML tumor cells entered a senescent‐like state after chemotherapy and that AML recurrence was promoted by this cellular senescence‐like restorative phenotype, which produced AML cells regardless of their stem cell status, leading to AML recurrence.^170^ Clinical trials have shown that the use of the BCL inhibitors venetoclax and azacitidine leads to a better prognosis than azacitidine alone in AML patients older than 75 years who had not previously received treatment.^166^ The use of cellular senescence scavengers is expected to improve the prognosis of patients with AML, especially elderly patients who are prone to recurrence.

### 5.2 Melanoma

Mutation‐mediated activation of BRAF is the earliest and most common genetic alteration in human melanoma, occurring in approximately 60% of melanoma cases, and BRAF mutations alone usually cause cellular senescence in melanocytes, but mutations in certain genes together with BRAF mutations can prevent cellular senescence.^171^ The PI3K pathway or depletion of the PTEN protein can lead to failure of cellular senescence caused by BRAF mutations, ultimately leading to malignant melanoma.^172^ Studies have shown that the activation of mTOR prevents cellular senescence caused by BRAF mutations, but this pathway does not appear to lead directly to the development of malignant melanoma.^173^ In addition to the role of BRAF, the role of c‐MYC in the regulation of senescence in melanoma is also important. One of the major functions of c‐MYC overexpression in melanoma progression is sustained inhibition of BRAF or RAS‐dependent cellular senescence, and when c‐MYC is lost or inhibited, it induces melanoma cellular senescence.^154^ Treatment of malignant melanoma was a thorny problem decades ago, and now, due to the development of immunotherapy and the emergence of BRAF suppression methods, these treatments have been greatly improved; however, some malignant melanomas are still prone to recurrence or even metastasis.^174^ These refractory malignant melanomas have been shown to be linked to cellular senescence.

Melanoma treatment showed good efficacy when administered in the early stage after the application of BRAF inhibitors (BRAFis), but drug resistance and recurrence sometimes occur when these agents are administered in a later stage, and the pathways involved in this drug resistance are diverse. We found that cellular senescence and the accompanying SASP may be involved in a special mechanism of melanoma resistance to chemotherapy.^175^ Experiments have demonstrated that in melanoma caused by BRAF mutations, the induction of cellular senescence by the BRAFi vemurafenib leads to the resistance of melanoma cells to this inhibitor.^176^ In vitro experiments demonstrated that the resistance of melanoma cells was reduced by clearing senescent cells: EGFR–SRC–STAT3 pathway activation was upregulated in BRAFi‐resistant melanoma cells. Dasatinib reduced the proliferation and invasion of these cells by inhibiting EGFR pathway activation, thereby overcoming drug resistance by targeting senescent cells.^177^

The effect of fisetin on melanocytes was analyzed in an in vitro three‐dimensional model system, and fisetin was found to promote tumor regression.^178^ Quercetin inhibited the growth, invasion and transfer potential of melanocyte lines.^179^ In addition, quercetin is metabolized by tyrosinase, which is expressed in melanocytes, and tyrosinases converted into anticancer compounds may show increased potency against specific melanomas.^180^ Piperone gold, a natural extract from the pepper plant (*Piper longum*), induces the apoptosis of melanoma cells.^181^

The benefits of senolytics in melanoma treatment are twofold. First, senolytics can clear senescent tumor cells to prevent the emergence of drug‐resistant cells. Second, they can be targeted against the antiapoptotic pathways in senescent cells that are associated with the development of secondary cancer and drug resistance.^182^ Intermittent doses of BRAFi, for example, have been shown to be particularly effective in the treatment of certain drug‐resistant melanomas,^183^ and these melanoma patients may benefit from alternating BRAFi treatments with the recently described “one‐two punch” heterotherapy. This “one‐two punch” treatment strategy calls for tumor‐targeted therapy to induce cellular senescence followed by xenoassays to help clear senescent cells.

### 5.3 Pancreatic cancer

Pancreatic ductal adenocarcinoma (PDAC) is closely associated with RAS‐induced senescence. RAS mutations were the first oncogene shown to induce senescence and are also the most common oncogene in PDAC. The onset of pancreatic cancer is usually associated with bypassing cellular senescence. When RAS is mutated alone, cells undergo cellular senescence. When K‐RAS mutation is accompanied by pancreatic injury, cells with K‐RAS mutation can proliferate under the action of IL‐33, but there is no cellular senescence. It has also been verified in fibroblasts that K‐RAS mutations can prevent cellular senescence accompanied by inactivation of p19ARF, p53,^184^ Rb, and p16ink4a.^4^ The former explains why pancreatitis patients are prone to cancer, while the latter implies that pancreatic cancer development is the result of a specific combination of mutations. When enough mutations cannot be compensated for, they are often intercepted by cellular senescence. In 2011, pancreatitis shown to cause pancreatic cancer by inhibiting OIS ^185^. Moreover, inhibiting specific tumor genes induced cellular senescence in a subset of mutant pancreatic cancer cells, particularly when MYC expression was suppressed. Loss of Rb in the pancreas has indeed been proven to prevent the cellular senescence caused by K‐RAS and cause cancer.^152^

Pancreatic cancer treatment is a global challenge, accounting for 2.5% of cancer patients worldwide in 2018 and accounting for 4.5% of cancer‐related deaths.^186^ Scientists are working to study the tumor microenvironment in pancreatic cancer patients in search of highly heterogeneous treatments. Tumors are often accompanied by extensive vascularization, but pancreatic cancer is characterized by few blood vessels; therefore, antiangiogenic therapy for pancreatic cancer has almost completely failed.^187^ A strategy based on normalization of blood vessels in pancreatic cancer is a new research focus, and immune sensitization in pancreatic cancer has been studied, with senescent cells showing a proangiogenic effect.^188^ In 2017, Lowe et al. exploited hyposensitivity and hypovolemia in PDAC immune therapy to actively age cancer cells and promote SASP factor release, thus leveraging the SASP to promote tumor vascularization and increase PDAC blood vessels, thus allowing more effective delivery of chemotherapeutic drugs. While promoting the penetration of cancer cell immune cells, such as T cells, into the lesion site of PDAC and improving PD1 sensitivity, this study presents an example of the combined use of cellular senescence inducers and immunomodulatory drugs to improve cancer treatment efficacy.^189^

### 5.4 Breast cancer

Breast cancer commonly manifests as breast hyperplasia caused by mutations in the RAS oncogene, which leads to malignant tumor formation after the cells escape the cellular senescence barrier.^190^ In addition, the common oncogene DcR2 has shown interesting characteristics in breast cancer, where it can induce G1‐stage to S‐stage stagnation, causing cellular senescence in cancer cells, which is generally low in general cancer but high in highly aggressive cancers.^191^ Cellular senescence, which is based on a patient's molecular pathology type, is an aspect that should be seriously considered in the development of personalized treatments for breast cancer. With a threefold increase in recurrence rates in patients with cellular senescence‐related p53/p14 double‐positive breast cancer compared with that in p53/p14 double‐negative patients, a fourfold increase in the recurrence rates in patients with P53/P16 double‐positive breast cancers, and a threefold increase in all‐cause‐related mortality, p53/p16 may be targets for obtaining valid bio‐clinical information for predicting the prognosis of breast cancer patients.^192^

Triple‐negative breast cancer (TNBC) is typically treated with combination chemotherapy. However, patients with TNBC often develop resistant, metastatic disease after they initially respond to chemotherapy. Chemotherapeutic resistance can develop through multiple mechanisms, including the induction of a transient growth arrest state known as TIS.^98^ Therapeutic cellular senescence is a heterogeneous manifestation of the clearance of TIS‐associated SASP factors, such as CXCL11, a static tumor growth‐promoting chemokine that has been shown to effectively reduce TNBC resistance and induce relapse, making it a potential target for TNBC therapy.^113^ The application of tyrosine kinase inhibitors for the treatment of HER2‐positive patients causes obvious tumor cellular senescence, and notably, the cellular senescence caused by these inhibitors is not related to p16 or p21. However, cellular senescence may be a potential pathway for resistance to HER2 inhibitors.^99^

Although cellular senescence easily leads to tumor resistance and recurrence, metastasis, and invasion can be inhibited by artificially promoting tumor cellular senescence. For example, the cellular senescence protein associated with the RANK protein in breast cancer is a typical two‐face protein. Before cancer occurs, the RANK protein can induce cellular senescence in the breast epithelium, which prevents malignant proliferation of the mutated breast epithelium, thus inhibiting the occurrence of cancer. However, after cancer occurs, cellular senescence of stromal cells induced by the RANK protein promotes stem cell accumulation in breast cancer cells, thus promoting metastasis and recurrence.^92^ One study used the PARP inhibitor ABT‐888 to induce the senescence of breast cancer cells and inhibit tumor growth.^94^ These findings revealed that inhibiting the AKT/ERK pathway with desicamabanin blocked the proliferation of TNBC cells and inhibited tumor growth, metastasis, and invasion. Notably, the therapeutic effect of norcantharidin completely prevented NF‐κB pathway activation, which may indicate that the effect of the AKT/ERK pathway on TNBC deserves further investigation.^133^

### 5.5 Lung cancer

There is evidence that lung cancer patients younger than the age of 50 years have a high incidence of targeted genome changes (such as EGFR mutation, ALK, or ROS1 fusion or ERBB2 insertion). Some evidence suggests that loss of c‐MYC plays a key role in the cellular senescence of small cell lung cancer.^153^ In contrast, other cancer‐causing driver genes, such as K‐RAS mutation, BRAF V600E, and MET exon 14 skipping, have been found in older lung adenocarcinoma patients. OIS in lung cancer remains to be explored. Chronic obstructive pulmonary disease (COPD) is a known risk factor for lung cancer. Understanding the relationship between COPD and cellular senescence could shed light on lung cancer development. This association was first noted when Holz discovered senescent cells in the tracheal tissue of COPD patients,^135^ and a series of subsequent studies revealed different cellular senescence phenotypes in endothelial cells, type I alveolar epithelial cells, and even immune cells.^193^ Subsequently, scientists, as represented by Krtorica, reported that senescent cells in COPD patients promoted the occurrence and development of lung cancer cells via SASP factors, and cellular senescence was shown to be an important factor that causes COPD, with PTEN mutations being particularly critical for cellular senescence and the occurrence of COPD.^194^ The absence of the PTEN phosphatase leads to activation of the PI3K/mTOR pathway and secretion of a large amount of SASP factors, which promote cellular senescence and chronic inflammation in bronchi, thereby driving the occurrence of COPD and accelerating the cellular senescence of proximal cells, forming a vicious cycle.^195^ Hence, senolytics that clear senescent cells or mTOR inhibitors that hinder PI3K/mTOR pathway activation can play a therapeutic role in lung cancer patients, including those with COPD.

The survival of mice with lung adenocarcinoma was successfully improved by targeting uPAR with CAR‐T cells to clear senescent cells.^137^ This study demonstrated not only the broad potential of CAR‐T cells but also the possibility of using senolytics for cancer treatment. The BCL‐XL inhibitor ABT263 also removed therapy‐induced senescent cells caused by lung cancer chemotherapy, and Saleh et al.^196^ preliminarily confirmed that ABT‐263 inhibited lung cancer tumor growth, prevented tumor recurrence, and improved patient prognosis. In addition, in response to the immune evasion characteristics of lung cancer cells during immunotherapy, the immunogenicity of lung cancer cells can be altered through cellular senescence induction, for example, lung cancer cells undergoing cellular senescence express high levels of the lung‐specific antigen LUNX.^197^

Senolytic agents have shown promise for use in predicting the effectiveness of lung cancer treatment, especially for radiation‐induced pulmonary fibrosis. Radiation‐induced senescence is a very common treatment for cancer, and radiation‐induced pulmonary fibrosis is an important cause of poor prognosis.

### 5.6 Prostate cancer

Loss of PTEN can induce prostate cell senescence, but loss of PTEN combined with loss of p53 can cause prostate cancer.^160^ During prostate tumorigenesis, TIMP acts as a key regulator that mediates prostate cancer metastasis after PTEN loss. TIMP can mediate the secretion of SASP components in senescent prostate cancer cells. TIMP activation leads to reduced production of SASP factors, thereby inhibiting the metastasis of prostate cancer. However, when TIMP and PTEN are both lost, prostate cancer cells usually metastasize.^198^

Prostate cancer is a typical cancer whose incidence increases with age, and its most well‐known type is benign prostatic hyperplasia. The relationship between prostate cancer and cellular senescence is unclear, and similar to other cancer cells, senescent prostate stromal cells secrete SASP factors in large quantities to promote the occurrence and development of cancer. These cells also secrete the most highly secreted factor, typically IL‐6. Activation of IL‐6 clearly leads to prostate carcinogenesis.^199^ However, whether the use of IL‐6 inhibitors increases survival in patients in the clinic remains to be determined.^200^ This lack of clarity of its effects may be related to the multiple functions of IL‐6. That is, IL‐6 is not simply a carcinogenic stimulus. In contrast, it plays an inhibitory role in the metastasis of specific prostate cancers. The STAT3 pathway can induce the cellular senescence of prostate cancer cells and thus inhibit cancer metastasis.^201^ Interestingly, most prostate cancer patients exhibit STAT3 expression, and STAT3 activation has been detected in numerous patients with metastatic prostate cancer.^202^ Moreover, inhibiting STAT3 in stat3‐positive patients with prostate cancer that had metastasized to bone stopped cancer progression.^203^ These findings may suggest that studying the role of STAT3 in inhibiting or activating the growth and metastasis of prostate cancer is worthwhile. Notably, this is not the only evidence suggesting that STAT3 can inhibit tumors. Is the dual‐edged nature of STAT3 derived from its ability to induce prostate cancer cellular senescence? In addition, the carcinogenic behavior of IL‐6 is complex. Without sufficient clinical evidence to support the use of IL‐6 antagonists, deeply analyzing targeted SASP factor similar to secreted IL‐6 and identifying their carcinogenic mechanisms to determine their use as therapeutic targets (e.g., with mTOR/PI3K inhibitors) will likely be better approaches than targeting IL‐6.

The role of cellular senescence caused by cancer treatment in prostate tumors cannot be ignored. Studies have shown that during the cellular senescence process caused by tumor treatment, the sensitivity of senescent tumor cells to BCL‐xl inhibitors increases significantly, and notably, the inhibition of cellular senescence in cells with increased sensitivity to BCL‐xl inhibitors is limited to those whose effects are mediated via ADP ribose polymerase 1 (e.g., senescent cells caused by DNA damage induced by ARP). Cellular senescence caused by the androgen antagonist enzalutamide was not among these inhibitors, and piperlongumine, a senescent cell scavenger, enhanced cellular senescence,^204^ suggesting that even the same patient with the same cancer can present different cellular senescence phenotypes when challenged with different stressors and that the presence or absence of androgen receptors is a decisive factor for the cellular senescence of prostate cancer cells.

## 6 CHALLENGES

Research on cellular senescence has lasted approximately one‐half century, and many theories on the mechanisms of cellular senescence have emerged, most of which are constantly changing and improving. Several views have been revised. Researchers of cellular senescence still face two challenges: identification of the gold standard marker and identification of senolytics.

All the abovementioned theories indicate the various types of cellular senescence, to date, we are still trying to determine how senescence influences cancer. A large number of markers of cellular senescence in cancer are currently available; however, there is no set of markers that specifically define all senescent cells, which poses some obstacles to the definition of senescent cells in different cancer under different conditions. A set of markers that specifically and exclusively indicates cellular senescence is needed, and identifying this marker is the greatest challenge. Currently, SA‐β‐gal is the most commonly used marker, but it cannot be used to identify all senescent cells; therefore, a more suitable new marker is needed. The National Institutes of Health has invested in research to identify markers of cellular senescence and create an atlas of senescent cells, and this agency expects to obtain a gold standard that can be used to explore and define cellular senescence better.^205^

Senescent cells are common but have unique effects on every cancer patient, and no high‐performance and low‐toxicity senolytics are available for use in clinical practice. ABT‐263 may be the most popular senolytic agent in the laboratory, it stably and effectively removes senescent cells, but clinical trials have revealed its serious adverse effects. In the case of immunotherapy, certain cellular senescence epitopes are often expressed on nonsenescent cells, which has led to doubts about their safety. Recently, D plus Q therapy at the Mayo Clinic has attracted considerable attention, but the final success of its application has yet to be determined. In fact, reliable animal models for promoting research on senescent cells are lacking. Neither the previously established P16‐expressing mice nor the recently generated p21CIP1 mice may properly represent the characteristics of human senescent cells.^9^, ^206^ Animal models that recapitulate the phenotypes of senescent human cells are needed to promote the clinical translation of senolytics.^207^ We anticipate that this breakthrough will soon occur.

## AUTHOR CONTRIBUTIONS

Liang Ma, Jie Yu, and Zhi Yang drafted the manuscript. Liang Ma, Yidian Fu, Xiaoyu He, and Zhi Yang contributed to manuscript revision. Shengfang Ge and Renbing Jia polished the manuscript and gave useful suggestions. Ai Zhuang, Zhi Yang, and Xianqun Fan played key role in editing, supervision, and funding acquisition. All authors have read and approved the final manuscript.

## CONFLICT OF INTEREST STATEMENT

The author(s) declare that they have no conflict of interests.

## ETHICS STATEMENT

Not applicable.

## Data Availability

Not applicable.
